# Editorial: Microbiota: A Consequential Third Wheel in the Mosquito-Pathogen Relationship

**DOI:** 10.3389/fmicb.2021.811880

**Published:** 2022-01-18

**Authors:** Mathilde Gendrin, Guido Favia, Jeremy K. Herren

**Affiliations:** ^1^Microbiota of Insect Vectors Group, Institut Pasteur de la Guyane, Cayenne, French Guiana; ^2^Department of Insect Vectors, Institut Pasteur, Université de Paris, Paris, France; ^3^School of Biosciences and Veterinary Medicine, University of Camerino, CIRM Italian Malaria Network, Camerino, Italy; ^4^International Centre of Insect Physiology and Ecology (icipe), Nairobi, Kenya

**Keywords:** microbiota, mosquito, host-microbe interactions, *Wolbachia*, symbionts, paratransgenesis, biological control

Mosquitoes are by far the most important vectors of human disease. There are hundreds of millions of cases of dengue annually, while Chikungunya and Zika have recently caused major outbreaks. Malaria remains a major driver of poverty in sub-Saharan Africa where it is responsible for about 400,000 deaths each year. In addition, about 50 million cases of lymphatic filariasis still occur annually.

The microbial communities harbored by mosquitoes have been the focus of great scientific interest since the discovery of their significant impact on disease transmission, via their influence on mosquito physiology and permissiveness to infection. In the 1990s and 2000s, the gut microbiota was found to limit parasitic infection in *Anopheles* malaria vector mosquitoes in experiments that used antibiotic treatments. It was subsequently demonstrated that *Wolbachia* endosymbionts could protect their insects hosts against viruses. In the 2010s, as high-throughput DNA sequencing became increasingly available to researchers, a more thorough description of the mosquito microbiota composition was generated and correlated to environmental or experimental parameters. Toward the end of the 2010s many more functional studies on mosquito/microbiota interactions were being carried out, and several countries had started to experiment with *Wolbachia*-infected mosquitoes as a public health measure to limit dengue transmission. These advances have culminated in a widespread appreciation that vector-pathogen interactions must be investigated in the context of a consequential third player, the microbiota.

In the article collection “Microbiota: A Consequential Third Wheel in the Mosquito-Pathogen Relationship,” we gathered state-of-the-art research on the microbiota of mosquitoes, including bacteria and eukaryotic microbes. This collection examines the interplay between three types of research in the field: functional characterization of host-microbe and microbe-microbe interactions, description of the microbiota system composition, and the design of microbiota-based tools to block disease transmission. These three aims are being advanced in parallel and are indeed interdependent, as functional characterizations identify specific attributes that can be used in the field if the system is better understood, whether functionally or taxonomically, while observations from applied research in the field bring novel questions to the basic research directions and a good description of the microbiota gives essential clues about how host-microbe associations work and how interventions may affect microbiota systems and what the consequences of this may be.

Historically, the first microbes used in the fight against vector borne diseases were entomopathogens, notably *Bacillus thuringiensis* var. *israelensis*, which is still widely used as a vector control tool against mosquitoes and other insect pests. Based on a screen for biopesticide activity in soil samples, Barbieri et al. have identified a new spore-producing bacterial isolate, *Brevibacillus laterosporus* SAM19, which is 10 times more efficient at killing *Aedes albopictus* than the reference *B. laterosporus* strain LMG15441 (Barbieri et al.). This biopesticide candidate may be used in a combination with other bacteria, such as *B. thuringiensis* var. *israelensis* and *Lysinibacillus sphaericus*, to avoid the evolution of resistance. Vector control may also be achieved using secondary metabolites produced by bacteria. For instance, prodigiosin produced by *Serratia marcescens* is known to have some larvicidal activity against several mosquito species as well as some antibacterial activity. Using prodigiosin-deficient bacteria, Heu et al. found that secondary metabolites, notably prodigiosin and/or serratamolide, participate in the virulence of *S. marcescens* in *Aedes aegypti* in adults and larvae, and in its antibacterial effect on several members of the mosquito microbiota. Their *in vitro* assays indicate that secondary metabolites are also essential in proteolytic and haemolytic activities (Heu et al.). Kulkarni et al. studied the impact of priming on infections by two strains of *Serratia* or *Enterobacter* in mosquitoes (Kulkarni et al.). They found that a preliminary oral infection by either strain had a protective effect on *Anopheles* mosquitoes after a subsequent septic challenge with the same bacterium. Their transcriptomic analysis discriminates between *Serratia*-infected mosquitoes that have been subjected to different priming conditions.

Moving beyond entomopathogens, members of the microbiota can also protect their host against infection by human pathogens, and therefore can decrease the vector competence of host mosquitoes. The most well-known example is that of *Wolbachia* endosymbionts, which naturally limit their host's susceptibility to infection by several viruses including Dengue and Zika in mosquitoes. These symbionts have been shown to manipulate mosquito reproduction to facilitate their spread through host populations. Indeed, among other phenomena, they can induce cytoplasmic incompatibility. This is essentially a male sterility phenotype that can only be rescued if the male mates with a female that carries the same *Wolbachia* strain. *Wolbachia*-colonized females, which can reproduce with any male, tend to have a higher fitness compared to non-infected females that are only compatible with non-infected males. Due to their capacity to invade mosquito populations and to reduce vector competence, these endosymbionts have been released at scale in last decade in several countries and are now considered a potentially transformative tool to fight arbovirus outbreaks. These *Wolbachia*-based control strategies are dependent on two of *Wolbachia's* phenotypes, protection against pathogens and cytoplasmic incompatibility. Notably, there are still many aspects of these two phenotypes that are not well-understood, including the nature of interactions between different strains in the same mosquito. Liang et al. investigated cytoplasmic incompatibility in the context of *Wolbachia* inter-strain competition (Liang et al.). They found that an infection with three strains of *Wolbachia* (wAlbA, wAlbB, and wMel) in *Ae. albopictus* led to a suppression of cytoplasmic incompatibility that is dependent on wAlbA. They also found differences between mosquito strains in the fitness cost of harbouring triple *Wolbachia* infections. Lu et al. investigated cellular aspects of DENV and ZIKV infection inhibition and showed that *Wolbachia* inhibits virion binding to mosquito Aag2 cells in a *Wolbachia*-density dependent manner, notably via the downregulation of dystroglycan and tubulin expression (Lu et al.).

Similar to *Wolbachia* in *Aedes* mosquitoes, *Microsporidia MB* is a fungi-related member of the *Anopheles* microbiota which has recently been proposed as a candidate tool to reduce malaria transmission, as it has been found to inhibit *Plasmodium falciparum* development in the mosquito gut. Focusing on how this symbiont is transmitted between mosquitoes, Nattoh et al. showed via co-housing experiments that horizontal transmission in *Anopheles arabiensis* occurs via mating (Nattoh et al.). *Microsporidia MB* can be detected in the seminal fluid of males, and can be vertically transmitted after a female is infected via mating. After screening several species sharing the habitats of *An. arabiensis*, the authors suggest that *Microsporidia MB* may be an *Anopheles*-specific symbiont. Another fungal member of the mosquito microbiota is the yeast *Wickerhamomyces anomalus*. The *W. anomalus-*host interaction was reviewed by Cappelli et al. Strains of this yeast produce a killer-toxin that is antimicrobial, since their approval by the European Food and Safety Authority these strains are now used in the agro-food sector to control mold and bacteria. Killer-toxin-producing *W. anomalus* is found in mosquitoes and has antiplasmodial activity, supporting its potential for use as a symbiotic-based control tool against malaria transmission. Other eukaryotes may also affect malaria transmission, but despite this the eukaryotic microbiota composition is still poorly described. While analysing the composition of the eukaryotic microbiota in *Anopheles* collected in Kenya, Burkina Faso, and Republic of Guinea, Cuesta et al. found that region of collection was the primary driver of microbiota differences and identified a new taxon in the *Ophryocystis* genus, which is highly prevalent in Kenyan mosquito samples (Cuesta et al.). As it belongs to Apicomplexa (the same phylum as *Plasmodium*), they suspect that interactions between both parasites may occur in *Anopheles* mosquitoes.

The bacterial microbiota has long been known to naturally limit infection of *Anopheles* mosquitoes by *Plasmodium*, yet this had not been tested in *Culex pipiens*, a vector for avian malaria. Martínez-de la Puente et al. observed that prevalence of *Plasmodium relictum* in saliva was higher in antibiotic-treated mosquitoes (Martínez-de-la-Puente et al.). They also detected a negative impact of the microbiota on mosquito life span in mosquitoes infected with *P. relictum*.

Another alternative route to using microbes for transmission blocking involved genetically-engineering them to inhibit infection of their mosquito host by parasites or viruses. This approach, called paratransgenesis, has been applied to several bacterial species including *Asaia* sp. and has been shown to efficiently limit *Plasmodium* infection in laboratory-reared *Anopheles*. Grogan et al. studied whether they could engineer an improved excretion of antiplasmodial effectors by *Asaia* sp. (Grogan et al.). They identified several novel secretion signals, including two which more efficiently excrete proteins and induce a higher level of antiplasmodial inhibition than their initial paratransgenetic strain.

Interactions between microbes will impact the success of any microbe-based approach applied to mosquitoes. For example, competitive interactions have been observed between *Wolbachia* and other members of the mosquito microbiota. Scolari et al. investigated the composition of the bacterial microbiota in *Ae. albopictus* and report correlations between *Wolbachia* and within-sample diversity (Scolari et al.). They also observed that developing larvae affect the microbial communities in their breeding water, corroborated with changes in pH and solutes. Conversely, environmental conditions during larval development, including microbiota composition and diet or larval density, have been found to impact larvae and even to have long-lasting effects during mosquito adulthood. MacLeod et al. more specifically examined how the amount of larval food affects mosquitoes and their microbiota (MacLeod et al.). They found that food abundance during larval development not only positively affects adult size, but also microbiota abundance and, though to a lesser extent, microbiota composition. Martinson and Strand used larvae colonised with a controlled microbiota to further investigate the impact of diet and microbiota composition on larval development (Martinson and Strand). They found that diet and microbiota both affect development success. Notably, microbiota composed of seven taxonomically-diverse bacteria better supports larval development than microbiota composed of each single bacterium even when larvae are provided a rich fish-food diet.

Last but not least, Gabrieli et al. reviewed the trilogy between the mosquito immune system, the microbiota and transmitted pathogens (Gabrieli et al.). They focus on the basic understanding of the interactions between the microbiota and the mosquito immune system before describing how the microbiota can be used to limit disease transmission via paratrangenesis or via the use of *Wolbachia*. They also contextualize this review with a section devoted to other insect vectors, including tsetse and sandflies.

Together, this article collection gathered studies focused on a diversity of mosquito microbe interactions, which reflects how the mosquito microbiome field is moving forward on many fronts. Through these studies and others, we have begun to understand the complexity of microbial communities and have even started to attribute certain functions to specific microbial members. In addition, these findings are advancing the prospect of symbiont-based control strategies (and paratransgenic control strategies). In the longer term, researchers in this field will hope to build on these studies to understand the precise causes and consequences of microbiota shifts and find ways to use the mosquito microbiota community or specific members to efficiently control vector borne diseases.

## Author Contributions

MG: writing—original draft. MG, GF, and JH: writing—review and editing. All authors contributed to the article and approved the submitted version.

## Funding

MG was supported by the French Government's Investissement d'Avenir program, Laboratoire d'Excellence Integrative Biology of Emerging Infectious Diseases (grant no. ANR-10-LABX-62-IBEID) by Agence Nationale de la Recherche (ANR, France) funding (MosMi grant no. ANR-18-CE15-0007 and PILGRIM grant no. ANR-20-CE35-0002-02). JH was supported by Open Philanthropy (SYMBIOVECTOR Track A) and the Bill and Melinda Gates Foundation (INV0225840). The International Centre of Insect Physiology and Ecology (*icipe*) receives support from the UK's Foreign, Commonwealth & Development Office (FCDO), Swedish International Development Cooperation Agency (Sida), Swiss Agency for Development and Cooperation (SDC), the Federal Democratic Republic of Ethiopia, and the Government of Kenya. GF was supported by the University of Camerino (grant no. FAR 2019) and by Italian Ministry for Research (MUR) (grant no. Prin, 2015JXC3JF).

## Conflict of Interest

The authors declare that the research was conducted in the absence of any commercial or financial relationships that could be construed as a potential conflict of interest.

## Publisher's Note

All claims expressed in this article are solely those of the authors and do not necessarily represent those of their affiliated organizations, or those of the publisher, the editors and the reviewers. Any product that may be evaluated in this article, or claim that may be made by its manufacturer, is not guaranteed or endorsed by the publisher.

